# Integrative Modeling of Multiomics Data for Predicting Tumor Mutation Burden in Patients with Lung Cancer

**DOI:** 10.1155/2022/2698190

**Published:** 2022-01-20

**Authors:** Jun Wang, Peng Chen, Mingyang Su, Guocheng Zhong, Shasha Zhang, Deming Gou

**Affiliations:** ^1^Shenzhen Key Laboratory of Microbial Genetic Engineering, Vascular Disease Research Center, College of Life Sciences and Oceanography, Guangdong Provincial Key Laboratory of Regional Immunity and Diseases, Carson International Cancer Center, Shenzhen University, Nanhai Ave 3688, Shenzhen, 518060 Guangdong, China; ^2^Department of Hematology and Oncology, Shenzhen University General Hospital, Shenzhen University Clinical Medical Academy, Xueyuan Ave 1098, Shenzhen, 518055 Guangdong, China

## Abstract

Immunotherapy has been widely used in the treatment of lung cancer, and one of the most effective biomarkers for the prognosis of immunotherapy currently is tumor mutation burden (TMB). Although whole-exome sequencing (WES) could be utilized to assess TMB, several problems prevent its routine clinical application. To develop a simplified TMB prediction model, patients with lung adenocarcinoma (LUAD) in The Cancer Genome Atlas (TCGA) were randomly split into training and validation cohorts and categorized into the TMB-high (TMB-H) and TMB-low (TMB-L) groups, respectively. Based on the 610 differentially expressed genes, 50 differentially expressed miRNAs and 58 differentially methylated CpG sites between TMB-H and TMB-L patients, we constructed 4 predictive signatures and established TMB prediction model through machine learning methods that integrating the expression or methylation profiles of 7 genes, 7 miRNAs, and 6 CpG sites. The multiomics model exhibited excellent performance in predicting TMB with the area under curve (AUC) of 0.911 in the training cohort and 0.859 in the validation cohort. Besides, the significant correlation between the multiomics model score and TMB was observed. In summary, we developed a prognostic TMB prediction model by integrating multiomics data in patients with LUAD, which might facilitate the further development of quantitative real time-polymerase chain reaction- (qRT-PCR-) based TMB prediction assay.

## 1. Introduction

Lung cancer is one of the most common malignancies worldwide, and it is the first leading cause of tumor-related mortality with an increasing incidence in recent years [1]. It was reported that 2.1 million new cases of lung cancer were diagnosed around the world in 2018, which accounted for 11.6% of all new cancer patients [2, 3]. Despite the improvements in chemotherapy and targeted therapy, the 5-year overall survival (OS) for patients with lung cancer remained poor [1, 4]. Nevertheless, immunotherapy, especially the application of immune checkpoint inhibitors (ICIs), had made a great breakthrough in the treatment of cancer and dramatically increased survival rate and quality of life for patients with lung cancer [5–9].

As the most successful representative of immunotherapy, programmed cell death-1/programmed cell death ligand-1 (PD-1/PD-L1) inhibitor had shown better performance over conventional chemotherapy in terms of OS, response rate, and progression-free survival (PFS) for the treatment of lung cancer [10, 11]. Furthermore, a large amount of clinical research had also demonstrated that immunotherapy alone or in combination with chemotherapy could be used for the first-line treatment of patients with metastatic lung cancer [12–15]. It was reported that patients with higher PD-L1 expression had better outcomes compared to patients with lower or no PD-L1 expression using anti-PD-L1 antibody clone 22C3 [16]. Unfortunately, only 10%-20% of non-small-cell lung cancer (NSCLC) patients have considerable curative effects, and most patients cannot benefit from immunotherapy [17–19]; therefore, biomarkers are urgently needed to rationalize the utilization of immunotherapy for patients.

Tumor mutation burden (TMB) emerged recently as a reliable biomarker that significantly correlated with immunotherapy efficacy across a wide spectrum of tumor types. TMB is defined as the number of somatic mutations per megabase (Mb) of the genome examined. Previous studies found that higher TMB was associated with improved objective response, durable clinical benefit, and PFS in NSCLC patients under immunotherapy [20]. It had been reported that PFS among stage IV patients with high TMB was significantly longer with PD-1/PD-L1 plus cytotoxic T-lymphocyte-associated protein 4 (CTLA-4) treatment than with chemotherapy [21]. Moreover, through analyzing 7,033 patients with different types of cancer, TMB was found to be a useful biomarker for predicting response of ICIs across different types of cancer, and higher TMB (highest 20% in each histology) was associated with better OS [22].

Whole-exome sequencing (WES) is considered the gold standard for evaluating TMB, but it is time-consuming and carries high cost [23]. Thus, targeted next generation sequencing (NGS) has been adopted as an alternative approach for predicting TMB. Memorial Sloan Kettering-Integrated Mutation Profiling of Actionable Cancer Targets (MSK-IMPACT) (468 genes) and FoundationOne companion diagnostic (CDx) (324 genes) are two extensively utilized targeted NGS methods, and both of them have been approved by the U.S. Food and Drug Administration (FDA) for clinical application. Despite the fact that targeted NGS is effective in predicting TMB, various problems arise for its routine clinical application, such as the limit of detection, germline mutation exclusion, and standard cutoff threshold determination [24]. Moreover, targeted NGS is still time-consuming and expensive compared with other clinical molecular tests [24]. In an effort to establish a simplified, cost-effective approach to predict TMB in patients with lung adenocarcinoma (LUAD), we intended to integrate a multiomics data to develop a predicting model for TMB.

**Figure 1:**
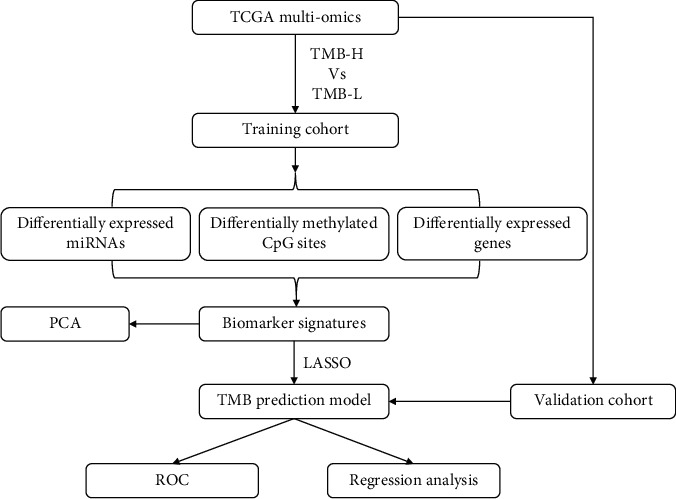
Flowchart of the analysis process in this study. TMB: tumor mutation burden; TMB-H: TMB-high; TMB-L: TMB-low; PCA: principal component analysis; LASSO: least absolute shrinkage and selection operator; ROC: receiver operating characteristic.

In this study (Figure 1), patients with lung adenocarcinoma (LUAD) in The Cancer Genome Atlas (TCGA) were first split into training and validation cohorts. Then patients in training cohort were divided as TMB-high (TMB-H) and TMB-low (TMB-L), and the differentially expressed genes, miRNAs, and differentially methylated CpG sites were identified. Subsequently, a multiomics TMB prediction model (TPM) involving expression profiles of selected genes, miRNAs, and methylation profiles of CpG sites was established. Finally, patients from the validation cohort were used to verify the performance of TPM.

## 2. Materials and Methods

### 2.1. Multiomics Dataset Acquisition from TCGA

Somatic mutation profiles of 567 samples, gene expression profiles of 594 samples, DNA methylation profiles of 507 samples, and miRNA expression profiles of 495 samples were obtained from TCGA database using either GDC tool (https://portal.gdc.cancer.gov/) or TCGAbiolinks R package (Supplementary Table S1) [25, 26]. The somatic mutation profiles (mutation annotation format, MAF) were processed by Mutect software. Missense mutations, nonsense mutations, splice-site mutations, frameshift insertions, frameshift deletions, in-frame insertions, or in-frame deletions identified in the samples were regarded as positive mutations. Gene expression profiles of 594 samples were annotated through g:Profiler website [27] and normalized using the scale method in limma package [28]. The low abundance profiles were eliminated. DNA methylation profiles were annotated using IlluminaHumanMethylation450kanno.ilmnl12.hg19 R package. Quality control for DNA methylation profiles was conducted through minfi R package to eliminate certain CpG sites [29], in which the single-nucleotide polymorphisms (SNPs) existed [30], multiple mapping to human reference genome was found [31], and the methylation information of any samples was not available. In addition, CpG sites located in sex chromosomes were excluded for analysis [32]. miRNA expression profiles of 495 samples including 450 samples from LUAD tissue and 45 samples from matched normal lung tissue in TCGA database were downloaded from the University of California Santa Cruz (UCSC) Xena database (https://xena.ucsc.edu/public). Then, the miRNA expression profiles were transformed into reads per million (RPM), and miRNAs expressed in more than 10% of patients with LUAD were extracted. The clinical information of 522 patients with LUAD from TCGA database was obtained using TCGAbiolinks R package [25], which covered id, age, gender, tumor stage, state, weight, Body Mass Index (BMI), alcohol history, height, days to last follow-up, years smoked, and race (Table 1).

### 2.2. TMB Calculation and Classification of Patients

Somatic mutation profiles were processed by Mutect software, and the identified somatic mutations, including base substitution, deletions, and insertions, were filtered according to the following criteria: (1) the minimum sequencing coverage for mutations should be greater than or equal to 10; (2) the variant allelic fraction should be greater than or equal to 5%. TMB was calculated as the total count of somatic mutations identified divided by 38 Mb, which is the length of exons in human genome. According to previously reported cutoff threshold of 10 in patients with LUAD [21, 33], patients were divided into TMB-H (TMB ≥ 10) and TMB-L (TMB < 10). Density plot of TMB-distribution for all patients with LUAD and boxplot of correlation between tumor stage and TMB was drawn by ggplot2 R package.

### 2.3. Tumor-Infiltrating Immune Analysis

Tumor-infiltrating immune analysis was performed through Tumor Immune Estimation Resource (TIMER) tool [34]. The estimated abundances of six immune infiltrates (B cells, CD4(+) T cells, CD8(+) T cells, neutrophils, macrophages, and dendritic cells) were compared between TMB-H and TMB-L patients.

### 2.4. Multiomics Analysis between TMB-H and TMB-L Patients

Differentially expressed genes between TMB-H and TMB-L patients in training cohort were identified through limma R package with -log10 adj.*p* value > 2 (adj.*p* value < 0.01) and log2 FoldChange > 3 [28] and then illustrated in volcano plot and heatmap by ggplot2 and pheatmap R package, respectively. Differentially expressed miRNAs between TMB-H and TMB-L patients in training cohort were identified through limma R package with -log10 adj.*p*-value > 1.30 (adj.*p*-value < 0.05) and log2 FoldChange > 0.35 [28] and then illustrated in volcano plot and heatmap by ggplot2 and pheatmap R package, respectively. In addition, target genes of the differentially expressed miRNAs were searched and analyzed through miRWalk website tool (http://mirwalk.umm.uni-heidelberg.de/) [35]. Differentially methylated CpG sites between TMB-H and TMB-L patients in training cohort were identified through limma R package with -log10 adj.*p* value > 1.30 (adj.*p* value < 0.05) and log2 FoldChange > 0.15 [28] and then illustrated in volcano plot and heatmap by ggplot2 and pheatmap R package, respectively.

### 2.5. Functional Enrichment Analysis

We first converted gene symbols into ENTREZ ID via org.Hs.eg.db R package, and then Gene Ontology (GO) and Kyoto Encyclopedia of Genes (KEGG) analysis of differentially expressed genes were implemented using ggplot2, enrichplot, and clusterProfiler R packages [36]. Meanwhile, GO and KEGG enrichment analysis were conducted for target genes of differentially expressed miRNAs using the same method described above.

### 2.6. Construction of TPM

We constructed 4 possible prediction biomarker signatures: gene signature (45 genes), miRNA signature (45 miRNAs), CpG site signature (45 CpG sites), and multiomics signature (15 genes + 15 miRNAs + 15 CpG sites) using differentially expressed genes, miRNAs, and differentially methylated CpG sites between TMB-H and TMB-L patients in the training cohort. Next, the least absolute shrinkage and selection operator (LASSO) logistic regression model analysis was performed to select the optimal biomarker signature for predicting TMB through glmnet R package [37]. The predictive performance for each biomarker signature was evaluated by lambda.min and matched area under curve (AUC). Finally, differentially expressed or methylated genes, miRNAs, and CpG sites identified with nonzero regression coefficients were used to construct the TPM. The TPM score was calculated using the regression coefficients from LASSO analysis to weigh the expression or methylation of the chosen biomarkers. The validation cohort was used to evaluate the performance of the TPM through assessing the predicting sensitivity, specificity, positive predictive value (PPV), negative predictive value (NPV), and AUC.

### 2.7. Principal Component Analysis (PCA)

Differentially expressed genes, miRNAs, and differentially methylated CpG sites identified through LASSO analysis were used to perform PCA. Expression or methylation profiles of the genes, miRNAs, and CpG sites were extracted from each patient, and ggfortify R package was utilized to conduct the PCA.

### 2.8. ROC Analysis

ROC curve analysis was conducted using pROC R package to investigate the performance of TPM in predicting TMB [38].

### 2.9. Correlation Analysis and Regression Analysis

Correlation between TPM score and TMB was analyzed by cor.test R function with the two-side Pearson's method. Samples were plotted in two-dimensional plots with the TPM score and TMB value. Regression analysis between TPM score and TMB was performed using lm R function.

## 3. Results

### 3.1. TMB-Based Division of Patients with LUAD

**Figure 2:**
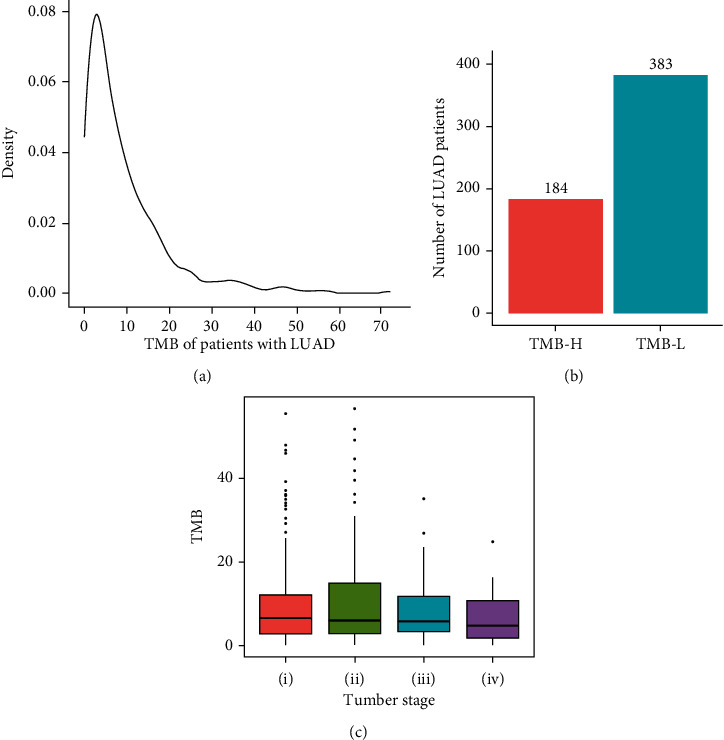
Division of patients with LUAD into TMB-H and TMB-L subgroups. (a) The distribution of TMB in patients with LUAD; (b) the number of TMB-H and TMB-L patients with LUAD; (c) the distribution of TMB across different tumor stages. TMB: tumor mutation burden; LUAD: lung adenocarcinoma; TMB-H: TMB-high; TMB-L: TMB-low; OS: overall survival.

**Figure 3:**
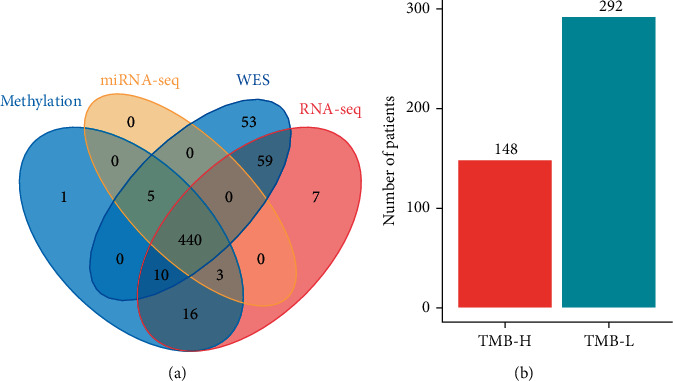
Multiomics data obtained from TCGA for patients with LUAD. (a) 440 patients with LUAD were found having coupled WES, DNA methylation, RNA-seq and miRNA-seq data; (b) 148 patients were classified as TMB-H and 292 patients were classified as TMB-L. WES: whole-exome sequencing; TMB-H: TMB-high; TMB-L: TMB-low.

WES data of tumor tissue from a total of 567 patients were acquired from TCGA-LUAD database, and clinical characteristics of the patients were summarized (Table 1); the mean age of the patients was 65.8, among which 242 individuals were males and 280 individuals were females (Table 1). TMB was calculated as the number of somatic mutations identified per megabase (Mb) in tumor tissue of each patient. It was found that most of patients with LUAD had a TMB ranging from 0 to 40 (Figure 2(a)). According to cutoff threshold of TMB = 10, 184 patients were classified as TMB-H and 383 patients were classified as TMB-L (Figure 2(b)). The TMB-H and TMB-L patients were found evenly distributed in different tumor stages as expected (Figure 2(c)). Furthermore, tumor samples from 440 patients were found also having RNA-seq, miRNA-seq, and DNA methylation datasets (Figure 3(a), Supplementary Table S1), among which 148 patients belong to the TMB-H group and 292 patients belong to the TMB-L group (Figure 3(b)). Patients were then randomly split into a training cohort (70%, 103 TMB-H patients vs. 204 TMB-L patients) and a validation cohort (30%, 45 TMB-H patients vs. 88 TMB-L patients) without overlap for developing a multiomics model to predict TMB.

### 3.2. Landscape of Tumor-Infiltrating Immune Cells in Patients with LUAD

**Figure 4:**
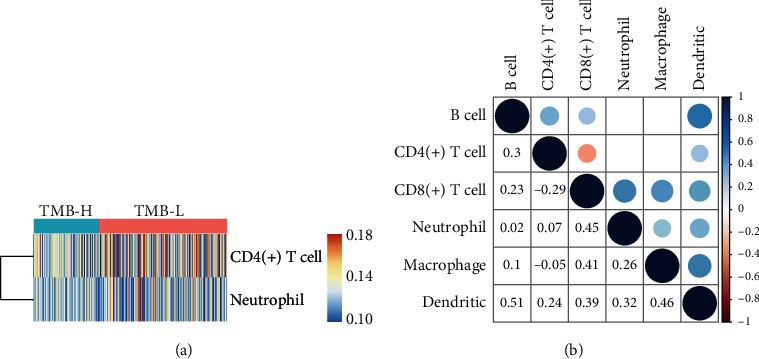
The landscape of tumor-infiltrating immune cells in TMB-H patients and TMB-L patients. (a) Relative proportions of infiltrating immune cells in TMB-H patients and TMB-L patients; (b) correlation matrix of all the proportions of 6 detected immune cell types. TMB-H: TMB-high; TMB-L: TMB-low.

Proportions of different tumor-infiltrating immune cells between TMB-H patients and TMB-L patients were calculated and summarized (Figure 4(a), Supplementary Figure S1), in which the abundance of CD4 (+) T cells (*p* = 0.030) showed more abundant density in TMB-L patients compared with TMB-H patients, whereas the B cell, CD8 (+) T cell, dendritic cell, macrophage cell, and neutrophil cell had similar density between TMB-H patients and TMB-L patients (Supplementary Figure S1, Figure 4(a)). Meanwhile, correlations among different tumor-infiltrating immune cell types were moderate or weak (Figure 4(b)). These results suggested that TMB might be associated with the abundance of CD4 (+) T cell in patients with lung cancer.

### 3.3. Multiomics Analysis of Transcriptome, miRNAome, and Methylome between TMB-H and TMB-L Patients

**Figure 5:**
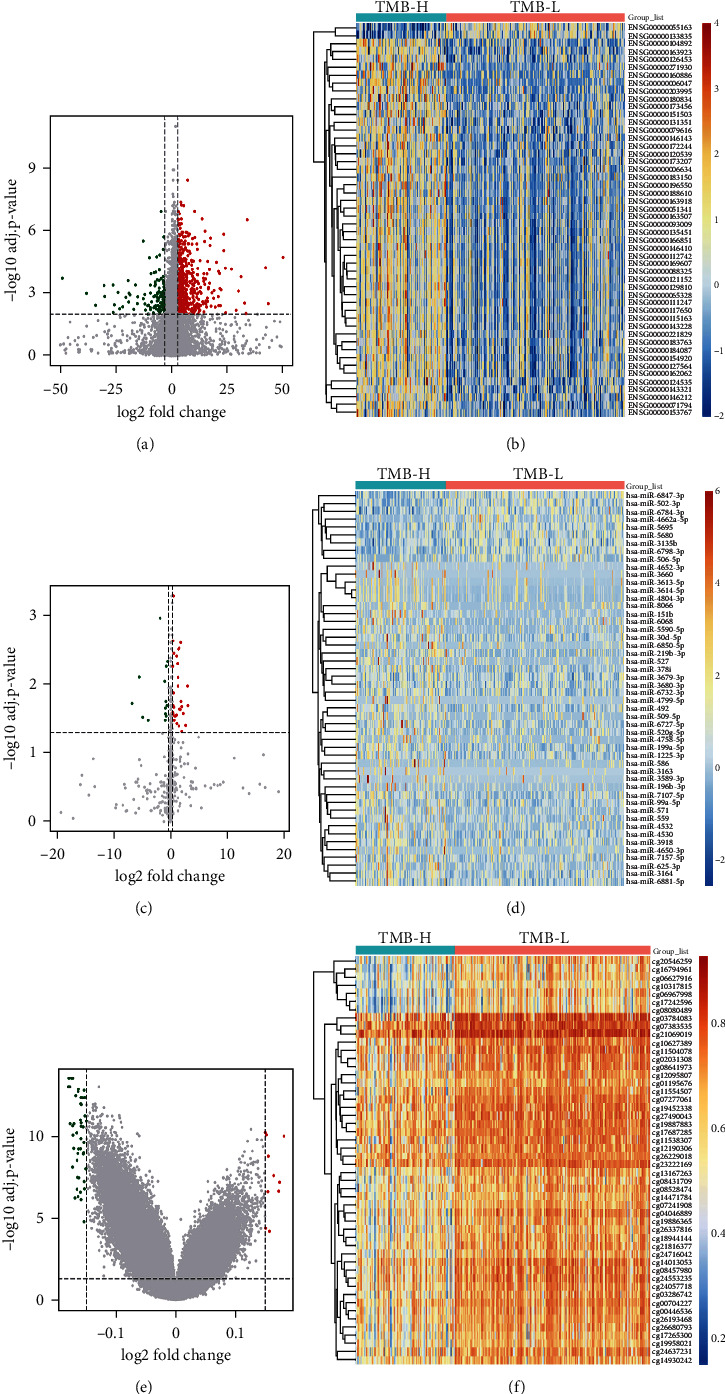
Characterization of the top50 differential expressed genes, miRNAs, and differential methylated CpG sites between TMB-H and TMB-L patients. Volcano plot showed the differentially expressed genes (a) and miRNAs (c) or differentially methylated CpG sites (e) between TMB-H and TMB-L patients. The red dots represent upregulated genes, miRNAs, or hypermethylated CpG sites; the blue dots represent downregulated genes, miRNAs, or hypomethylated CpG sites; the black dots represent genes, miRNAs, or CpG sites with no significantly differential expression or methylation. Hierarchical clustering heatmap of differentially expressed genes (b) and miRNAs (d) or differentially methylated CpG sites (f) between TMB-H and TMB-L patients. Orange indicates the upregulated genes, miRNAs, or hypermethylated CpG sites; blue indicates the downregulated genes, miRNAs, or hypomethylated CpG sites. TMB-H: TMB-high; TMB-L: TMB-low.

To explore differences in a tumor microenvironment between TMB-H and TMB-L patients, differentially expressed genes, miRNAs, and differentially methylated CpG sites between TMB-H patients and TMB-L patients in the training cohort were identified. In summary, 480 genes and 36 miRNAs were upregulated in TMB-H patients, whereas 130 genes and 14 miRNAs were downregulated in TMB-H patients (Figures 5(a)–5(d)). Moreover, 10 CpG sites were hypermethylated and 48 CpG sites were hypomethylated in TMB-H patients (Figures 5(e) and 5(f)). GO-enrichment analysis suggested that the differentially expressed genes were mainly involved in the biological processes including nuclear division, chromosome segregation, and organelle fission (Supplementary Figure S2A). KEGG pathway enrichment analysis suggested that the differentially expressed genes were mainly related to pyrimidine metabolism (Supplementary Figure S2B). These results demonstrated that differentially expressed genes might be correlated with carcinogenesis-related processes [39]. In addition, GO and KEGG enrichment analysis were also performed for the target genes of differentially expressed miRNAs, which were found to be mainly enriched in netrin-activated signaling pathway, DNA-binding transcription activator, and single-stranded RNA binding (Supplementary Figure S3A, Figure S3B). In addition, most of the differentially methylated CpG sites were found to locate in gene body regions (Supplementary Figure S4), and 5 CpG sites were found to locate in the TSS1500 (sequence region from -200 to -1500 nt upstream of the transcription start site) and TSS200 (sequence region -200 nt upstream of the transcription start site) region of genes (Supplementary Figure S4, Supplementary Table S2).

### 3.4. Machine Learning-Based Construction of TMB Prediction Model

**Figure 6:**
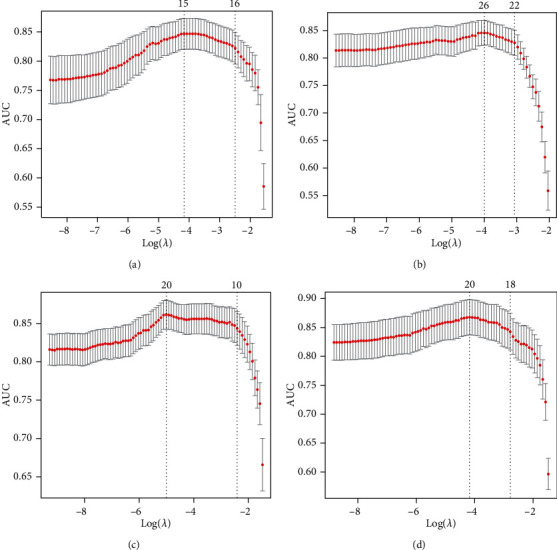
LASSO regression analysis for 4 possible prediction biomarker signatures. 10-fold cross-validation in LASSO regression analysis for gene signature (a), miRNA signature (b), CpG site signature (c), and multiomics signature (d). LASSO: least absolute shrinkage and selection operator; AUC: area under curve.

**Figure 7:**
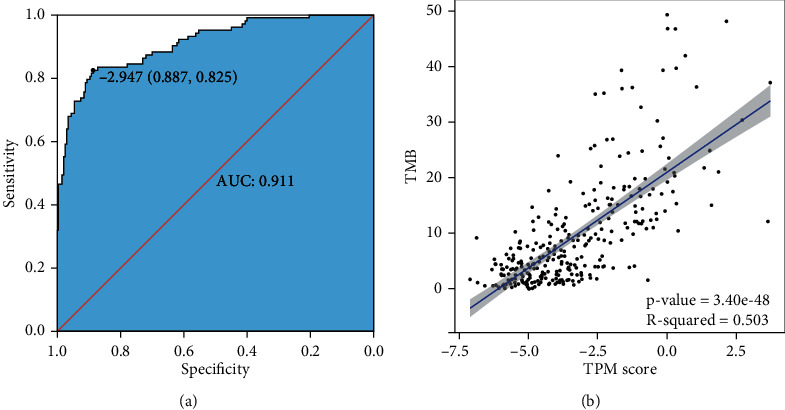
The performance of TPM in the training cohort. (a) ROC analysis of the TPM score in the training cohort; (b) the TPM score is highly correlated with TMB. TPM: TMB prediction model; AUC: area under curve.

To develop TPM based on the differences identified through multiomics analysis, we firstly generated 4 possible prediction biomarker signatures including gene signature, miRNA signature, CpG site signature, and multiomics signature, which were composed of expression profiles of top 45 differentially expressed genes, top 45 differentially expressed miRNAs and top 45 differentially methylated CpG sites between TMB-H and TMB-L patients, respectively (Supplementary Table S3). To further compare predicting efficacy of the four signature, we implemented LASSO logistic analysis to select the optimal signature from training cohort. The optimal biomarkers for the 4 prediction signature were obtained with nonzero regression coefficients (Figure 6, Table 2), and as a result, the multiomics signature with maximum measure (0.868) was selected as the optimal biomarker signature for predicting TMB (Figure 6(d), Table 2). PCA using the shrunk multiomics signature suggested that TMB-H patients and TMB-L patients could be separated obviously (Supplementary Figure S5). Based on the multiomics signature, we finally constructed TPM by weighing expression or methylation of the genes, miRNAs, and CpG sites through regression coefficients from the LASSO analysis (Table 3). The TPM was showed as the following math formula: TPM score = −1.555696454∗cg02031308 − 0.939485314∗cg03286742 − 0.532855695∗cg04046889 − 1.603385472∗cg12095807 − 1.171295176∗cg16794961 − 1.341848062∗cg24553235 + 0.203290638∗YBX2 + 0.000323171∗HLTF + 0.355814358∗KLC3 + 0.017454209∗WRNIP1 + 0.010739241∗CKS1B + 0.013056543∗RNF26 + 0.039397451∗ZYG11A + 0.582628142∗hsa − miR − 571 + 3.954182602∗hsa − miR − 586 + 0.068239671∗hsa − miR − 151b + 0.000724033∗hsa − miR − 378i + 0.25824073∗hsa − miR − 6727 − 5p − 0.731679875∗hsa − miR − 502 − 3p − 0.007119299∗hsa − miR − 6798 − 3p. The AUC of the constructed TPM in the training cohort was 0.911 showing its superior predictive accuracy (Figure 7(a)). Besides, the *p* value of a two-side *t*-test was 3.40*e* − 48 between TPM score and TMB (Figure 7(b)), which suggested that TPM score was highly correlated with TMB in patients with LUAD.

### 3.5. Evaluation of the Predicting Accuracy of TPM in the Validation Cohort

**Figure 8:**
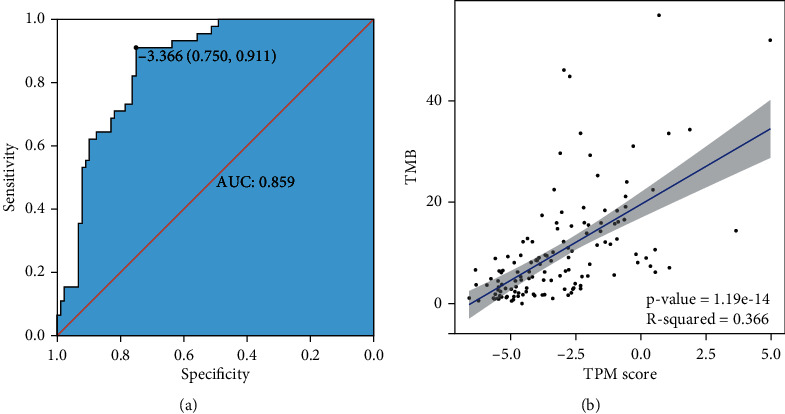
The performance of TPM in the validation cohort. (a) AUC of ROC analysis was 0.859 showing the great predictive accuracy of TPM; (b) the TPM score is highly correlated with TMB with *p* value = 1.19*e* − 14. TPM: TMB prediction model; AUC: area under curve.

To evaluate the predicting efficacy of TPM constructed in training cohort, expression or methylation profiles of genes, miRNAs, and CpG sites in patients from validation cohort were used as input parameters for calculating TPM score. According to the threshold of -3.366, 41 patients from the TMB-H group were predicted as TMB-H, and 66 patients from the TMB-L group were predicted as TMB-L. In summary, the TPM has a sensitivity of 0.911, a specificity of 0.750, and an accuracy of 0.805 in predicting TMB in the validation cohort, and the PPV was 0.651 and NPV was 0.943 (Supplementary Table S4). ROC analysis revealed the AUC of the TPM in validation cohort was 0.859 (Figure 8(a)), and *p* value of the two-side *t*-test was 1.19*e* − 14 between the TPM score and TMB (Figure 8(b)). These results suggested that the TPM performed relatively high TMB-predicting accuracy in an independent validation cohort.

## 4. Discussion

Immunotherapy has been demonstrated particularly successful in NSCLC treatment and is being adopted as a first-line treatment option worldwide [12, 21, 40]. Nevertheless, only a small portion of unselected patients can benefit from immunotherapy [24, 41]. Therefore, biomarkers for patient selection become important to achieve effective therapy. TMB has been recognized as the effective prognostic biomarker in NSCLC patients according to the results from numerous clinical trials [21, 22, 42, 43]. Although targeted NGS has been proved to be an alternative approach of WES for the prediction of TMB, the accuracy and simplicity of targeted NGS remain challenging as various parameters should be taken into consideration [44]. In this study, we developed a mathematic multiomics model that could precisely predict TMB in patients with LUAD, and the prediction accuracy of the model was validated in an independent cohort with high sensitivity and specificity. Furthermore, as the input parameter in this model includes expression profiles of 7 genes, 7 miRNAs, and the methylation profiles of 6 CpG sites, which could be obtained through quantitative real time-polymerase chain reaction (qRT-PCR). This model paved the way for further development of the simplified qRT-PCR-based clinical assay for TMB prediction.

The tumor microenvironment refers to the network of cells and structures surrounding a tumor cell, and it consists of immune cells, mesenchymal cells, endothelial cells, extracellular matrix (ECM) molecules, and inflammatory mediators [45]. High TMB indicates the presence of more neoantigens in tumor microenvironment, which promotes the inflammatory response and results in the alteration of transcriptomic and epigenetic changers [45]. It has been proved that gene expression signatures in the tumor microenvironment were associated with the prognosis in NSCLC [46–49]. In agreement with previous studies, the differentially expressed genes between TMB-H and TMB-L patients identified in this study were found to enrich in the immune-related damaged DNA binding, nuclear division, nuclear chromosome segregation, organelle fission, single-stranded DNA binding, ribonucleoprotein complex binding, and pyrimidine metabolism (Supplementary Figure S2) [50–54]. The 7 genes used in constructing TPM might be involved in carcinogenesis; for instance, Y box binding protein 2 (YBX2) was differentially expressed between different subtypes of breast cancer and was one of RNA processing factors which contribute to subtype-specific splicing [55]. Meanwhile, it was found that LINC00958 promoted cell proliferation and migration in oral squamous cell carcinoma through the miR-627-5p/YBX2 axis [56]. Moreover, it was reported that the wild type alleles of kinesin light chain 3 (KLC3) Lys751Gln were significantly correlated with greater smoking intensity, and genetic variations may influence the progression of lung cancer [57]. In addition, the expression of CDC28 protein kinase regulatory subunit 1 (CKS1B) in lung cancer cells developed the chemoresistance through the Hsp90 and MEK1/2 pathway [58].

miRNA expression in tumor microenvironment plays a crucial role in mediating and controlling several immune and cell interactions and convolutes in the regulation of immune checkpoints, PD1 and PD-L1 [59]. It was reported that a 25 miRNA-based signature classifier could predict the TMB level with high accuracy [60]. A cluster of highly expressed miRNA including hsa-miR-492, hsa-miR-498, and hsa-miR-320 were found to be correlated with tumorigenesis of retinoblastoma [61]. Moreover, the invasion, proliferation, and migration of cervical cancer cells were found to be promoted by hsa-miR-6727-5p, which might play an important role in cervical cancer progress [62]. In this study, we mapped the differentially expressed miRNAs between TMB-H and TMB-L patients to their target genes, and enrichment analysis of the target genes suggested that DNA-binding transcription activator, single-stranded RNA binding, MAP kinase activity, and glycerolipid metabolism that related to lung cancer were affected in a tumor microenvironment. The 7 miRNAs used in constructing the TPM in this study include hsa-miR-571, hsa-miR-586, hsa-miR-151b, hsa-miR-378i, hsa-miR-6727-5p, hsa-miR-502-3p, and hsa-miR-6798-3p, among which hsa-miR-378i and miR-502-3p were demonstrated to be important for colorectal cancer carcinoma metastasis [63, 64].

Changes in DNA methylation are one of the most important epigenetic alterations in a tumor microenvironment. A multicenter study in 15 hospitals suggested epigenomic profile based on a microarray DNA methylation signature (EPIMMUNE) could serve as an effective biomarker in predicting the outcomes of NSCLC patients treated with PD-1 inhibitors [65], and the FOXP1 could be a predictive biomarker for better-selecting patients to benefit with immunotherapy [65]. The CpG site signature also had a relatively high predictive performance (measure = 0.861) of TMB in our study, suggesting its great value in NSCLC prognosis. cg02849937 located in the TSS1500 region of C7orf13 and its expression level were negatively associated with promoter methylation using whole-genome integrative analysis [66]. In addition, cg27281030 is located in the TSS1500 region of NLRP12, which has been demonstrated regulate inflammation, and it is believed that hepatocellular carcinoma was negatively regulated by NLRP12 through suppression of inflammation and proliferation of hepatocytes [67].

Through multiomics analysis, we integrated gene/miRNA expression and DNA methylation data to reflect subtle alterations of the tumor microenvironment to precisely predict TMB for better prognosis of patients with LUAD in immunotherapy. Fragments per kilobase per million mapped reads (FPKM) of YBX2, HLTF, KLC3, WRNIP1, CKS1B, RNF26, ZYG11A, and RPM of hsa-miR-571, hsa-miR-586, hsa-miR-151b, hsa-miR-378i, hsa-miR-6727-5p, hsa-miR-502-3p, and hsa-miR-6798-3p as well as beta-value of cg02031308, cg03286742, cg04046889, cg12095807, cg16794961, and cg24553235 were extracted from RNA-seq, miRNA-seq and Illumina HumanMethylation450 BeadChip, respectively, for calculating TPM score. Although the FPKM, RPM, and beta value involved in the TPM were based on high-throughput sequencing or chip analysis, it is feasible to convert them to cycle threshold (Ct) value in qRT-PCR analysis and thus simplify the prediction of TMB by using benchtop qRT-PCR instrument. The conversion of FPKM in different samples to Ct values could be probably through comparison of the targeted gene expression to reference gene expressions, such as actin and eukaryotic elongation factor (eEF), which have relative consistent expression under different tumor microenvironment, and beta value of CpG sites could also be converted into Ct value though the quantitative MethyLight technology [68]. To our best knowledge, this is the first time to construct the TPM for patients with LUAD from multiomics view.

## 5. Conclusion

In summary, the present study developed a multiomics risk model with high specificity and sensitivity in predicting TMB for patients with LUAD and laid the base for establishing a more simplified and cost-effective TMB test assay. Nevertheless, this study was solely bioinformatics research, and clinical sample validation for the TPM had not been implemented. The training cohort and the validation cohort used in this study were relatively small in size and required further expansion to increase the accuracy.

## Figures and Tables

**Table 1:** Clinical information of 522 TCGA-LUAD patients.

Variables	Statistics
Gender	
Male (%)	242 (46.4%)
Female (%)	280 (53.6%)
Age	
80~89 (%)	30 (5.8%)
70~79 (%)	150 (28.7%)
60~69 (%)	146 (28.0%)
50~59 (%)	83 (16.0%)
40~49 (%)	25 (4.8%)
30~39 (%)	2 (0.4%)
Not reported (%)	86 (16.3%)
Race	
White (%)	393 (75.3%)
Black or African American (%)	53 (10.2%)
Asian (%)	8 (1.5%)
American Indian or Alaska native (%)	1 (0.2%)
Not reported (%)	67 (12.9%)
Status	
Alive (%)	334 (64.0%)
Dead (%)	188 (36.0%)
Tumor stage	
I (%)	279 (53.4%)
II (%)	124 (23.8%)
III (%)	85 (16.3%)
IV (%)	26 (5.0%)
Not reported (%)	8 (1.5%)

LUAD: lung adenocarcinoma.

**Table 2:** The performance of 4 optimal biomarker signatures obtained by LASSO regression analysis.

Biomarker signature	Optimal biomarkers	lambda.min	Measure
Gene	GTF2IRD1, FTSJ1, CHMP4B, KLC3, DMAC2, GIT1, SOHLH2, SYNGR3, SAP130, LRRC1, FN3KRP, POU4F1, ZNF526, KRT80, UBE2C, FOXE1, MEX3D, CIDECP1, PRR19, DHX16, FANCG, AC010632.1, AC019171.1	0.018	0.884
miRNA	hsa-miR-22-5p, hsa-miR-486-5p, hsa-miR-492, hsa-miR-561-5p, hsa-miR-151b, hsa-miR-3677-5p, hsa-miR-3923, hsa-miR-4425, hsa-miR-4434, hsa-miR-4536-5p, hsa-miR-4679, hsa-miR-5702, hsa-miR-6727-5p, hsa-miR-6858-5p, hsa-miR-7107-5p, hsa-let-7 g-3p, hsa-miR-136-3p, hsa-miR-155-3p, hsa-miR-371a-3p, hsa-miR-491-3p, hsa-miR-432-3p, hsa-miR-574-3p, hsa-miR-3074-3p, hsa-miR-3622b-3p, hsa-miR-3679-3p, hsa-miR-3150b-3p, hsa-miR-4639-3p, hsa-miR-4655-3p, hsa-miR-6798-3p, hsa-miR-6847-3p	0.017	0.734
CpG site	cg01862650, cg02031308, cg02916472, cg07184316, cg07729440, cg10120778, cg10488199, cg11002952, cg20151576, cg20297017, cg20671274, cg21827634, cg22773522, cg23049130, cg25841348	0.015	0.845
Multiomics	cg01862650, cg07729440, cg20671274, cg21827634, cg22773522, GTF2IRD1, FTSJ1, TTI1, CHMP4B, KLC3, HNRNPUL1, UBE2S, BCL2L12, SYNGR3, KRT80, FOXE1, AC006213.3, hsa-miR-22-5p, hsa-miR-492, hsa-miR-4536-5p, hsa-miR-6727-5p, hsa-miR-7107-5p, hsa-miR-136-3p, hsa-miR-3679-3p, hsa-miR-6816-3p	0.010	0.938

LASSO: least absolute shrinkage and selection operator: TMB: tumor mutation burden.

**Table 3:** Coefficient of each biomarker of multiomics signature in LASSO model analysis.

	Biomarkers	Coefficient
Multiomics	cg01862650	-1.818730262
	cg07729440	-6.256940647
	cg20671274	-0.911730577
	cg21827634	-3.867697524
	cg22773522	-2.460934084
	GTF2IRD1	0.039415926
	FTSJ1	0.012286235
	TTI1	0.042479337
	CHMP4B	0.009500278
	KLC3	0.454704618
	HNRNPUL1	0.015834511
	UBE2S	0.014978452
	BCL2L12	0.078730525
	SYNGR3	0.192724739
	KRT80	0.017563239
	FOXE1	0.011660062
	AC006213.3	0.175314579
	hsa-miR-22-5p	1.230322969
	hsa-miR-492	-0.185026622
	hsa-miR-4536-5p	7.457452192
	hsa-miR-6727-5p	0.560095048
	hsa-miR-7107-5p	-0.131067725
	hsa-miR-136-3p	0.93577811
	hsa-miR-3679-3p	-0.007961529
	hsa-miR-6816-3p	1.359432308

LASSO: least absolute shrinkage and selection operator.

## Data Availability

Somatic mutation profiles of 567 samples, gene expression profiles of 594 samples, DNA methylation profiles of 507 samples, and miRNA expression profiles of 495 samples were obtained from TCGA database.

## Supplementary Materials

Supplementary Materials Supplementary Table S1: summary of TCGA-LUAD data. Supplementary Table S2: five CpG sites located in the TSS1500 and TSS200 region of the genes. Supplementary Table S3: different biomarker signatures used in the training cohort. Supplementary Table S4: the performance of TPM in the validation cohort. Supplementary Figure S1: the differences in the abundance of tumor-infiltrating immune cells between TMB-H and TMB-L patients. Student's *t*-test was used; error bars indicated standard deviation; ^∗^*p* < 0.05. TMB-H: TMB-high; TMB-L: TMB-low. Supplementary Figure S2: GO and KEGG analysis for the differentially expressed genes. Supplementary Figure S3: GO and KEGG analysis for the target genes of differentially expressed miRNAs. Supplementary Figure S4: distribution of differentially methylated CpG sites in genes. Supplementary Figure S5: principal component analysis (PCA) for the multiomics signature after LASSO variable reduction.
