# Supplementary material for: Integrative Modeling of Multiomics Data for Predicting Tumor Mutation Burden in Patients with Lung Cancer
Source: Biomed Res Int. 2022 Jan 20;2022:2698190. doi: 10.1155/2022/2698190 (PMC8794677; doi:10.1155/2022/2698190)
Supplement: Supplementary Materials — Supplementary Table S1: summary of TCGA-LUAD data. Supplementary Table S2: five CpG sites located in the TSS1500 and TSS200 region of the genes. Supplementary Table S3: different biomarker signatures used in the training cohort. Supplementary Table S4: the performance of TPM in the validation cohort. Supplementary Figure S1: the differences in the abundance of tumor-infiltrating immune cells between TMB-H and TMB-L patients. Student's t-test was used; error bars indicated standard deviation; ∗p < 0.05. TMB-H: TMB-high; TMB-L: TMB-low. Supplementary Figure S2: GO and KEGG analysis for the differentially expressed genes. Supplementary Figure S3: GO and KEGG analysis for the target genes of differentially expressed miRNAs. Supplementary Figure S4: distribution of differentially methylated CpG sites in genes. Supplementary Figure S5: principal component analysis (PCA) for the multiomics signature after LASSO variable reduction. [file 2698190.f1.docx]

**Supplementary Data**

**Supplementary Table S1** Summary of TCGA-LUAD data

| **Data** | **Tumor** | **Normal** | **Total** |
| --- | --- | --- | --- |
| WES | 567 | 0 | 567 |
| RNA-seq | 535 | 59 | 594 |
| miRNA-seq | 450 | 45 | 495 |
| DNA methylation | 475 | 32 | 507 |
| Clinical information | 522 | 0 | 522 |

Tumor: According to the sample type codes of TCGA, the 14th and 15th digits of tumor sample barcode range from 01 - 09; Normal: According to the sample type codes of TCGA, the 14th and 15th digits of normal sample barcode range from 10 - 19.

**Supplementary Table S2.** Five CpG sites located in the TSS1500 and TSS200 region of the genes

| **CpG sites** | **Genes** | **Region** |
| --- | --- | --- |
| cg02849937 | C7orf13 | TSS1500 |
| cg02849937 | RNF32 | TSS1500 |
| cg27281030 | NLRP12 | TSS1500 |
| cg17463633 | LOC100130331 | TSS1500 |
| cg23179456 | ADCY4 | TSS200 |

TSS1500, sequence region from -200 to -1500 nt upstream of the transcription start site; TSS200, sequence region -200 nt upstream of the transcription start site.

**Supplementary Table S3.** Different biomarker signatures used in the training cohort

|  | **Counts** | **Biomarkers** |
| --- | --- | --- |
| Genes | 45 | YBX2, ZYG11A, KLC3, CENPA, CKS1B, TRAIP, MAP6D1, SGO1, HLTF, MCM10, MTFR2, WRNIP1, AC016877.3, RNF26, POC1A, PRIM2, EME1, NUF2, RPL39L, CYFIP2, HSD17B4, PKMYT1, BCL2L12, C5orf34, NEK2, GPR19, TEDC2, FANCG, TROAP, FAM72A, MASTL, LRRC42, RAD51AP1, TPX2, LY6K, HAUS8, HDGF, CDC45, FAM72B, NCAPD3, GTF2E1, PLK1, KIF22, CKAP2L, DBF4 |
| miRNAs | 45 | hsa-miR-378i, hsa-miR-571, hsa-miR-6732-3p, hsa-miR-6798-3p, hsa-miR-3614-5p, hsa-miR-7107-5p, hsa-miR-502-3p, hsa-miR-6727-5p, hsa-miR-492, hsa-miR-151b, hsa-miR-3660, hsa-miR-586, hsa-miR-3164, hsa-miR-520g-5p, hsa-miR-625-3p, hsa-miR-3613-5p, hsa-miR-30d-5p, hsa-miR-4804-3p, hsa-miR-99a-5p, hsa-miR-3163, hsa-miR-4758-5p, hsa-miR-5695, hsa-miR-3679-3p, hsa-miR-4799-5p, hsa-miR-199a-5p, hsa-miR-6847-3p, hsa-miR-559, hsa-miR-3918, hsa-miR-7157-5p, hsa-miR-5680, hsa-miR-6881-5p, hsa-miR-4532, hsa-miR-6784-3p, hsa-miR-3680-3p, hsa-miR-5590-5p, hsa-miR-527, hsa-miR-4662a-5p, hsa-miR-219b-3p, hsa-miR-5589-3p, hsa-miR-6850-5p, hsa-miR-6068, hsa-miR-4652-3p, hsa-miR-509-5p, hsa-miR-196b-3p, hsa-miR-506-5p |
| CpG sites | 45 | cg04046889, cg06967998, cg02031308, cg16794961, cg17242596, cg12095807, cg08641973, cg03286742, cg17265300, cg06627916, cg08080489, cg08457980, cg24553235, cg26337816, cg19886365, cg27490043, cg24637231, cg11554507, cg18944144, cg19452338, cg13167263, cg07277061, cg07241908, cg19958021, cg08528474, cg10627389, cg12190306, cg26229018, cg01195676, cg26680793, cg00446536, cg00704227, cg07383535, cg19887883, cg21816377, cg26193468, cg11504078, cg17687285, cg24057718, cg14013053, cg10317815, cg20546259, cg14930242, cg23222169, cg11538307 |

**Supplementary Table S4.** The performance of TPM in the validation cohort

|  | **Sensitivity** | **Specificity** | **PPV** | **NPV** | **Accuracy** | **AUC** |
| --- | --- | --- | --- | --- | --- | --- |
| Validation cohort | 0.911 | 0.750 | 0.651 | 0.943 | 0.805 | 0.859 |

TPM, TMB prediction model; PPV, positive predictive value; NPV, negative predictive value; AUC, area under curve.

**
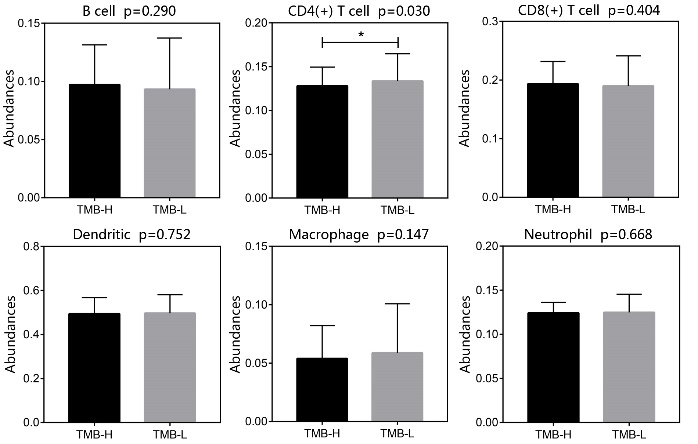
**

**Supplementary Figure S1** The differences in the abundance of tumor-infiltrating immune cells between TMB-H and TMB-L patients. Student’s t test was used, error bars indicated standard deviation, * p < 0.05. TMB-H, TMB-high; TMB-L, TMB-low.


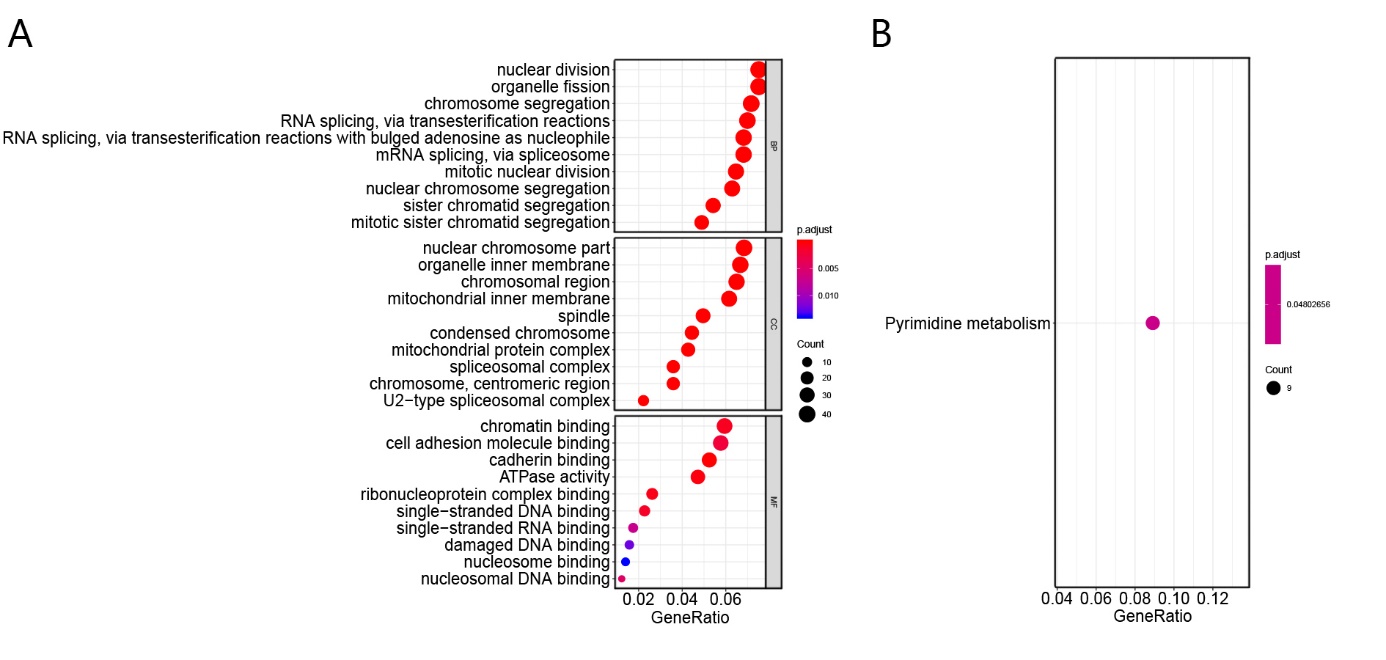


**Supplementary Figure S2** GO and KEGG analysis for the differentially expressed genes. (**A**) GO analysis for the differentially expressed genes between TMB-H patients and TMB-L patients; (**B**) KEGG analysis for the differentially expressed genes between TMB-H patients and TMB-L patients. GO, Gene Ontology; KEGG, Kyoto Encyclopedia of Genes and Genomes.

**
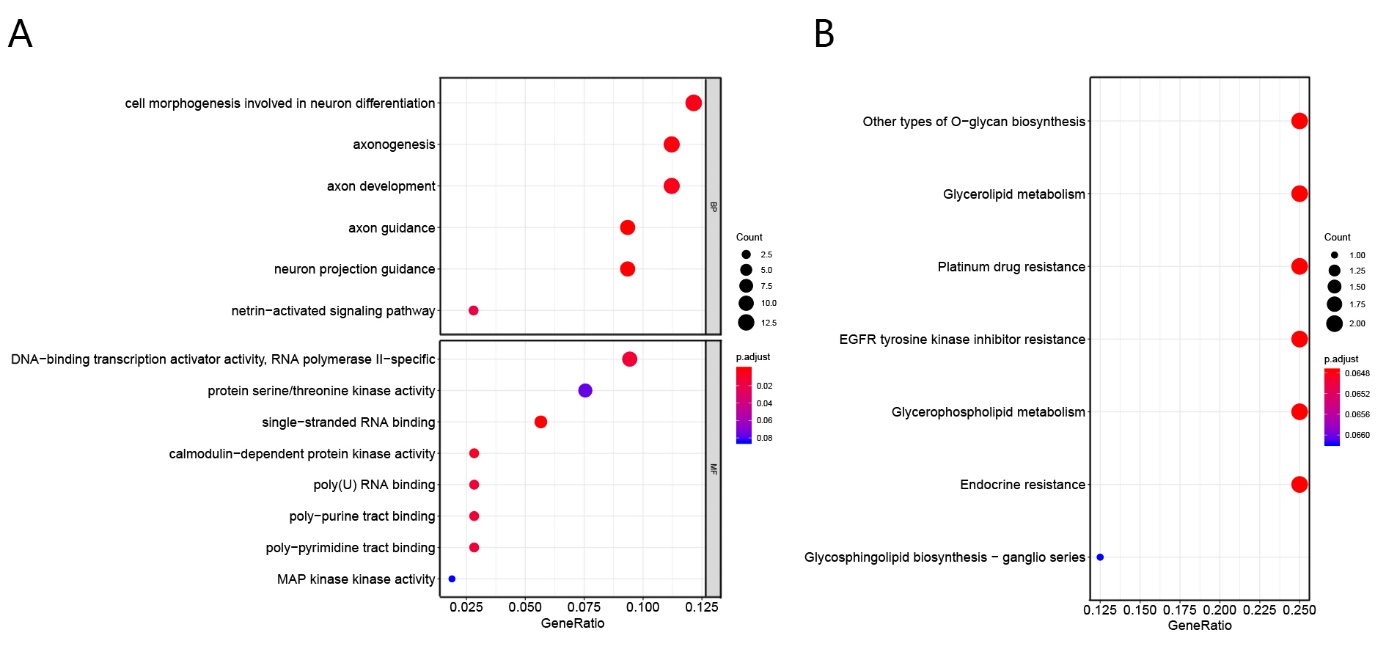
**

**Supplementary Figure S3** GO and KEGG analysis for the target genes of differentially expressed miRNAs. (**A**) GO analysis for the target genes of differentially expressed miRNAs between TMB-H patients and TMB-L patients; (**B**) KEGG analysis for the target genes of differentially expressed miRNAs between TMB-H patients and TMB-L patients. GO, Gene Ontology; KEGG, Kyoto Encyclopedia of Genes and Genomes.


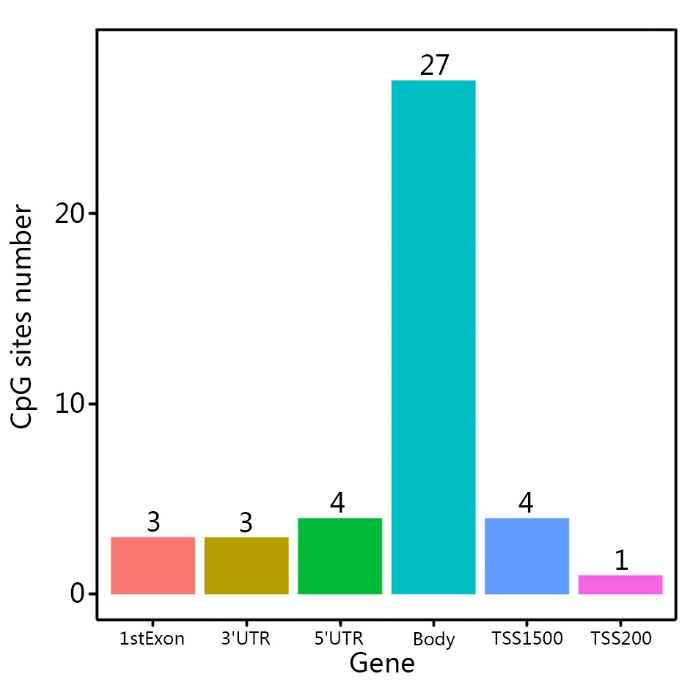


**Supplementary Figure S4** Distribution of differentially methylated CpG sites in genes. 1stExon, first expressed region; 3’UTR, 3’ untranslated region; 5’UTR, 5’ untranslated region; TSS1500, sequence region from -200 to -1500 nt upstream of the transcription start site; TSS200, sequence region -200 nt upstream of the transcription start site.


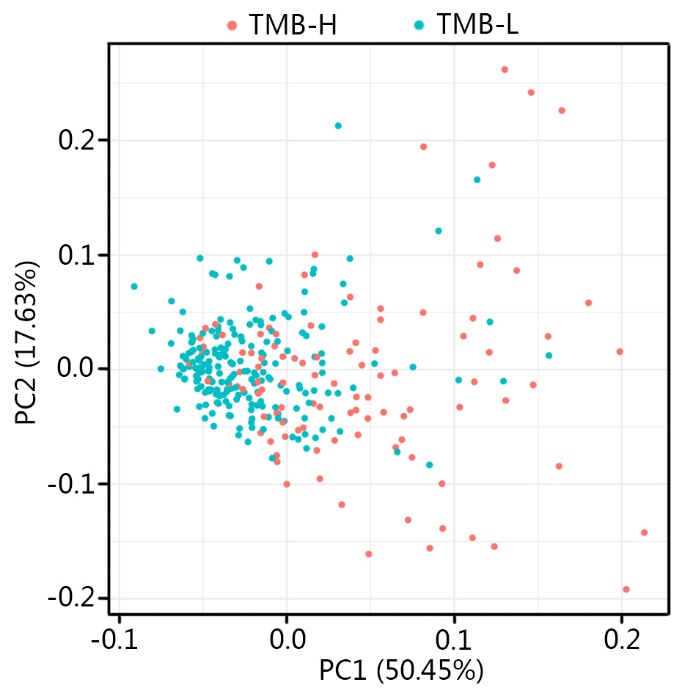


**Supplementary Figure S5** Principal component analysis (PCA) for the multi-omics signature after LASSO variable reduction.
